# Progression-related outcomes of teriflunomide in multiple sclerosis clinical trials

**DOI:** 10.3389/fneur.2026.1882453

**Published:** 2026-08-13

**Authors:** Farhad Mahmoudi, Nicole Baldwin, Ramon Flores Gonzalez, Solomon Nittala, Kottil Rammohan, Micheline McCarthy, Flavia Nelson

**Affiliations:** 1 Department of Neurology, University of Miami, Miami, FL, United States; 2 Essentia Health Neurology, Duluth Clinic, Duluth, MN, United States

**Keywords:** cognition, disability, DMT, MRI, MS, pro, teriflunomid

## Abstract

**Introduction:**

Multiple sclerosis (MS) is a chronic inflammatory disease of the central nervous system that leads to neurological disability, more pronounced in the progressive phase. Teriflunomide, an oral disease-modifying therapy, has shown potential neuroprotective effects beyond relapse reduction, including preservation of brain integrity and mitigation of disability progression. This review provides supportive evidence of the effects of teriflunomide on disability progression, cognition, and MRI markers of neurodegeneration in MS.

**Methods:**

PubMed/MEDLINE, Embase, Scopus, and Web of Science were searched to identify relevant studies evaluating the effects of teriflunomide on disability progression, cognition, and MRI markers of neurodegeneration in MS such as brain volume loss. Clinical trials, extension studies, comparator studies, and real-world observational cohorts were reviewed.

**Discussion:**

Teriflunomide demonstrated consistent effects stabilizing disability progression, maintaining cognitive performance, reducing brain volume loss and preserving gray and white matter integrity across multiple studies. Findings from various studies support its role in limiting disability progression. Proposed mechanisms include preservation of mitochondrial integrity, reduction of oxidative stress, and promotion of remyelination. Although higher-efficacy therapies may provide superior inflammatory control, teriflunomide may have an important role in the long-term management of MS.

**Conclusion:**

Teriflunomide is emerging as a valuable, multi-faceted contributor to the MS treatment landscape, with data supporting its role in preserving brain volume, slowing disability progression, and maintaining cognitive function. Additionally, its favorable long-term tolerability and minimal immunocompromise effects further support its value as a therapeutic option over the longer course in PwMS.

## Introduction

Multiple sclerosis (MS) is characterized by central nervous system (CNS) inflammation mediated demyelination, axonal transection and neurodegeneration, leading to accumulation of neurological disability (1). Relapse associated worsening (RAW) and progression independent of relapse activity (PIRA) are two terms which reflect our current understanding of disability accumulation, which drastically changed with the use of highly effective disease modifying therapies (DMT) and better control of relapse activity in the past decade or so. To date, PIRA remains challenging to understand and consequently treat. Despite being the current focus of industry sponsored trials of novel therapeutic interventions, disease progression is still affecting the majority of our MS population. A key MRI marker of MS progression is accelerated atrophy, specifically whole brain (WB), cortical gray matter (cGM) and thalamic, all of which can begin early and continue throughout the course of the disease (2). It is well known that rates of brain volume loss (BVL) in MS far exceed those of normal aging and have been linked to worsening physical disability and cognitive decline (3). Early treatment has been shown to have an effect on brain and thalamic volume loss but typically not reverting BVL to a normal rate. It is also well known that few available DMTs delay disease progression but that is not the focus of this review. There is, however, one DMT, teriflunomide, which has shown surprising results in recent clinical trials when used as a comparator vs. a novel DMT.

**Figure 1:**
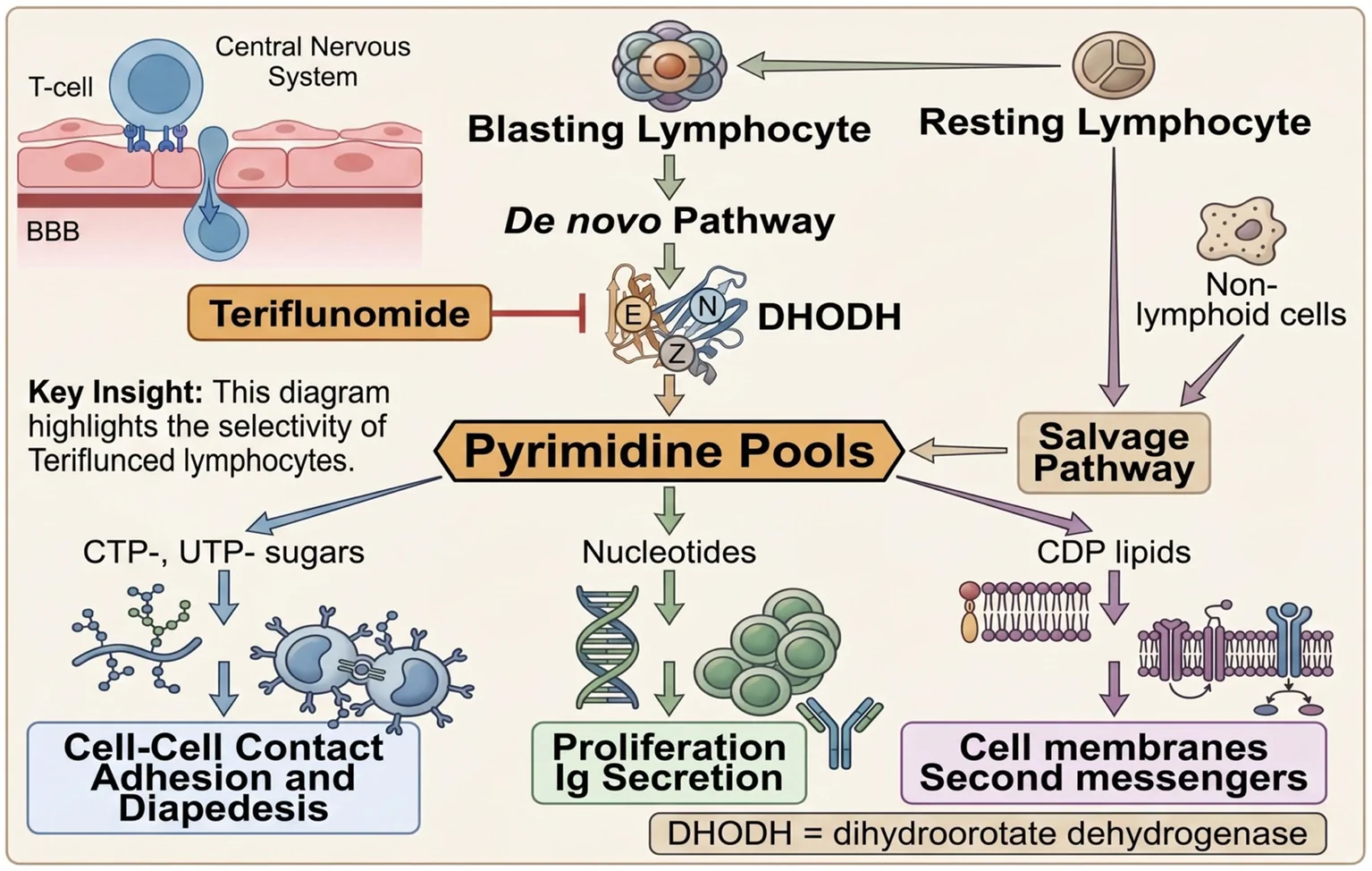
Teriflunomide blocks the *de novo* pathway of pyrimidine synthesis via a non-competitive and reversible inhibition of the mitochondrial enzyme, dihydroorotate dehydrogenase (DHODH), and inhibits the proliferation of active T- and B- lymphocytes and suppresses antibody production in the periphery. Additional mechanisms have also been proposed.

**Figure 2:**
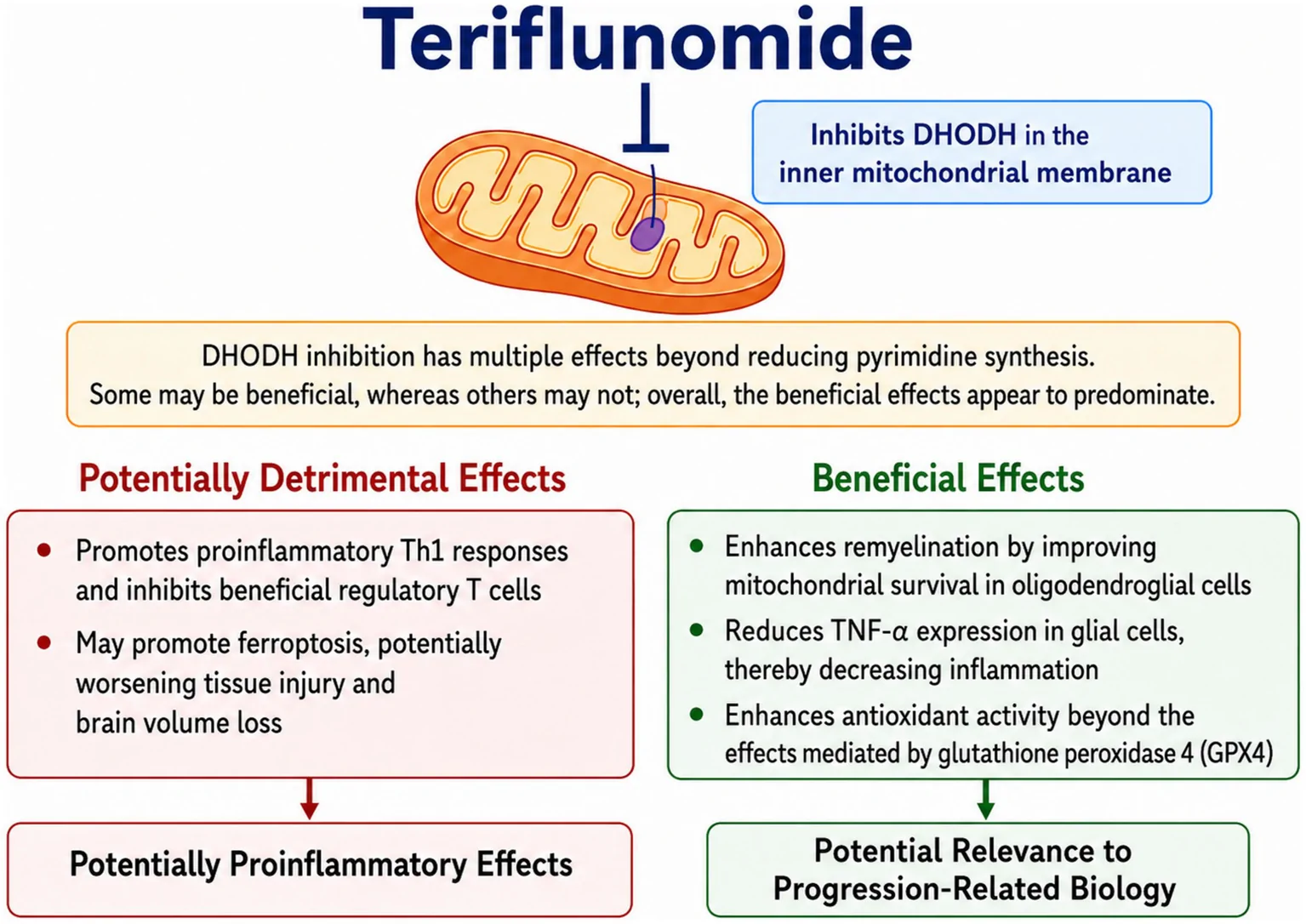
Desirable and undesirable effects of teriflunomide. DHODH, dihydroorotate dehydrogenase; GPX4, glutathione peroxidase 4; PIRA, progression independent of relapse activity; RAW, relapse-associated worsening; Th1, T helper 1; TNF-*α*, tumor necrosis factor-alpha; T-regs, regulatory T cells.

Teriflunomide is an oral DMT that selectively inhibits dihydroorotate dehydrogenase (DHODH), blocking *de novo* pyrimidine synthesis in proliferating lymphocytes (4). This cytostatic effect reduces T and B cell-mediated inflammation while sparing non-proliferating cells (4) (Figure 1). In addition, teriflunomide regulates mitochondrial function and cellular metabolism (5, 6) thought to play a role in MS progression. Within the CNS, teriflunomide may reduce inflammatory signaling, support mitochondrial integrity and promote repair processes, including remyelination (7). These beneficial effects may contribute to maintaining tissue integrity and mitigation of disease progression. However, teriflunomide may also have competing effects at the level of lymphocyte-mediated inflammation. Some mitochondrial effects may reduce regulatory T-cell function, promote a relative shift toward pro-inflammatory responses, and increase susceptibility to lipid peroxidation, which leads to ferroptosis (8, 9) (Figure 2). Overall, clinical and MRI data support that the net biological impact favors anti-inflammatory and neuroprotective mechanisms (Table 1).

**Table 1:** Mechanisms of action of teriflunomide and potential relevance to PIRA in MS.

Domain	Mechanism	Cellular target	Functional effect	Clinical relevance	Potential relevance to progression (PIRA)
Immune	Inhibition of de novo pyrimidine synthesis (4)	Proliferating lymphocytes	↓ lymphocyte proliferation (cytostasis)	Immunomodulation	↓ peripheral inflammatory activity
Immune	Inhibition of mitochondrial OXPHOS (6)	Affinity-dependent T cells	↓ expansion of activated T cells; restoration of T-cell repertoire	Immune rebalancing	May limit chronic immune-driven tissue injury
Immune	Interference with mitochondrial respiration; inhibition of mitochondrial Complex III (8)	FoxP3^+^ regulatory T cells (Tregs)	↓ Treg suppressive capacity; ↑ Th1 shift	Potentially pro-inflammatory effect	May contribute to sustained low-grade inflammation
CNS/glial	Shifts astrocytic metabolism toward glycolytic ATP production (5)	Astrocytes	↓ TNF-α secretion	Reduced neuroinflammation	May reduce CNS inflammation
CNS/repair	Restoration of mitochondrial integrity (7)	Oligodendrocytes	↑ myelin repair	Tissue preservation	Supports remyelination and limits axonal degeneration
Mitochondrial	Prevention of mitochondrial fragmentation and fission (39)	Neurons/peripheral nerves	↓ oxidative injury	Neuroprotection	↓ axonal loss and neurodegeneration
Mitochondrial	Antioxidant effect (38)	Peripheral nerve mitochondria	Protection against oxidative stress	Neuroprotection	Preserves neuronal integrity
Mitochondrial/cell death	DHODH inhibition → ↑ P53-ALOX15 signaling → ↑ lipid peroxidation (9)	Neurons	↑ susceptibility to ferroptosis	Potentially detrimental effect	May increase vulnerability to neurodegeneration under stress conditions

ALOX15, arachidonate 15-lipoxygenase; ATP, adenosine triphosphate; CNS, central nervous system; DHODH, dihydroorotate dehydrogenase; OXPHOS, oxidative phosphorylation; P53, tumor protein p53; PIRA, progression independent of relapse activity; TNF-α, tumor necrosis factor alpha; Tregs, regulatory T cells.

Multiple studies have examined whether teriflunomide affects key markers of MS progression, including brain atrophy, worsening disability, and cognitive decline. This article reviews the evidence from pivotal and recent trials, long-term extensions, supporting teriflunomide’s effects on clinical measures of disability progression, MRI metrics such as BVL, as well as other structural MRI changes in gray and white matter, and discusses how these data translate into disability outcomes and cognitive performance (Supplementary material).

### Literature search strategy

A targeted literature search of PubMed/MEDLINE, Embase, Scopus, and Web of Science was conducted through February 2026 to identify studies evaluating teriflunomide in MS. Search terms included “teriflunomide,” “multiple sclerosis,” “disability progression,” “confirmed disability worsening,” “progression independent of relapse activity (PIRA),” “brain atrophy,” “MRI,” “cognition,” “neuroprotection,” and “progressive multiple sclerosis,” along with related keywords. Eligible studies included randomized controlled trials, extension studies, *post hoc* analyses, observational cohorts, comparator trials, and mechanistic studies evaluating teriflunomide in relation to clinical, MRI, cognitive, and progression-related outcomes. Studies were selected based on their relevance to the objectives of this narrative review.

## Pivotal clinical trials of teriflunomide

### Effect on MRI metrics

In untreated patients, BVL (brain atrophy) occurs at an annual rate of approximately 0.5–1.35%, which is several times faster than the rate observed in healthy individuals (10). This accelerated atrophy reflects widespread neuroaxonal loss and is considered a sensitive and reproducible biomarker of disease progression (11). As such, BVL has become a key imaging endpoint in clinical trials evaluating the efficacy of DMTs in MS (12).

Brain atrophy outcomes in Phase III trials: The Phase III TEMSO study was the first large trial (~1,000 patients) to assess teriflunomide’s impact on MRI measures over 2 years in relapsing MS (RMS). In the original BVL analysis there was no statistically significant difference between groups; in a post-hoc re-analysis of TEMSO MRI data, teriflunomide slowed whole-brain atrophy compared to placebo. Teriflunomide 14 mg once daily significantly slowed brain atrophy. At year 2, BVL was lower with teriflunomide 14 mg vs. placebo (0.90% vs. 1.29%; 30.6% reduction, *p* = 0.0001). Importantly, both the 14 mg and 7 mg doses showed reduced BVL compared to placebo over the 108-week period. These results provide evidence that teriflunomide significantly reduces the rate of global BVL in RMS. Importantly, the effect was observed in patients with and without clinical disability progression during the trial, suggesting that teriflunomide’s impact on brain volume is consistent across different patient subsets (13). Teriflunomide reduced brain volume loss by about 30–40 percent compared to placebo over 2 years, which is considered clinically meaningful (13).

**Figure 3:**
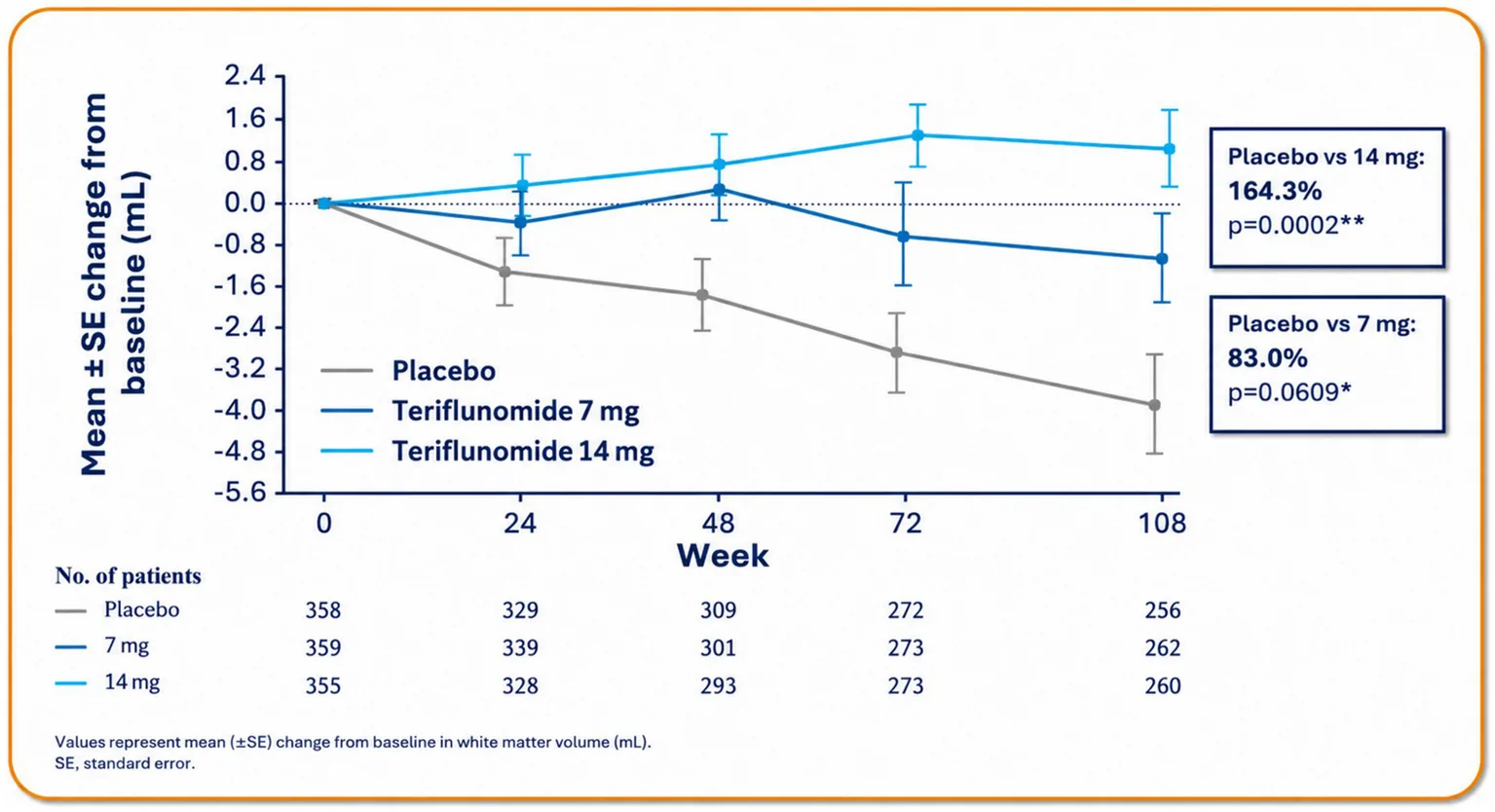
Mean (±SE) change from baseline in white matter volume over 108 weeks in patients receiving placebo, teriflunomide 7 mg, or teriflunomide 14 mg in the TEMSO trial. Adapted with permission from Wolinsky et al. (15).

Importantly, teriflunomide 14 mg also demonstrated an increase in white matter volume (mean change +1.23 mL vs. −4.07 mL in placebo), suggesting preservation of structural brain integrity beyond lesion-level effects (Figure 3). Additionally, a subgroup analysis of the 122 patients (5.4%) with active secondary progressive and progressive RMS (at screening) from TEMSO and TOWER (second phase III trial), showed similar efficacy and effect on MRI endpoint compared to the effect on the RMS analysis. Long-term Expanded Disability Status Scale (EDSS) data in these patients was shown to remain stable for up to 6 years (14). Other MRI endpoins showed a 67.4% lower accumulation in total lesion volume, along with a 76.7% reduction in T2-hyperintense and a 31.3% reduction in T1-hypointense lesion volume. There was also an 80.4% reduction in Gd-enhancing lesions per scan and a 69.4% reduction in unique active lesions compared to placebo. These benefits were sustained over time and consistent across multiple patient subgroups, regardless of age, sex, baseline lesion burden, or disease subtype. Together, these findings highlight teriflunomide’s ability to reduce inflammatory activity while also limiting tissue damage and preserving brain structure, which are critical elements in slowing MS progression (15).

Early intervention in CIS the TOPIC trial: Teriflunomide’s effects on brain atrophy extend to patients with clinically isolated syndrome (CIS). In the Phase III TOPIC trial, teriflunomide 14 mg significantly slowed whole-brain atrophy over the first 2 years of disease. At 12 months, the annualized rate of whole-brain atrophy was lower by 43% (*p* < 0.0001) versus placebo. This indicates that teriflunomide can preserve brain tissue even early in the disease process. Additionally, lower WB and cortical gray matter (cGM) atrophy in teriflunomide-treated CIS patients was accompanied by a lower risk of conversion to clinically definite MS (CDMS).

The analysis showed that each 1% decrease in WB volume was associated with a 47.3% increased risk of conversion to CDMS within 2 years and a 35.9% increase over a 5-year period. Accordingly, patients with the least atrophy in the first 2 years had a significantly reduced probability of CDMS compared to those with the most atrophy. These findings suggest that teriflunomide has the potential to alter the disease trajectory in early MS (16).

### Evidence of gray matter, white matter, and thalamic volume preservation

While BVLis a useful global measure, regional atrophy in MS, particularly in cGM and deep GM structures, has strong clinical relevance for both physical and cognitive outcomes (17). MS causes widespread damage in cortical and deep GM (e.g., thalamus). Teriflunomide’s impact on these specific tissue compartments has been investigated using advanced MRI analyses and real-world imaging studies.

Cortical gray matter: In the TOPIC trial, teriflunomide 14 mg significantly slowed cortical gray matter loss. At 12 months, the annualized rate of cGM volume loss was 61.4% lower with teriflunomide than with placebo. By month 24, teriflunomide reduced cortical atrophy’s rate by 40% (*p* = 0.042) compared to placebo. Similarly, an observational single-center study of 120 patients comparing teriflunomide vs. dimethyl fumarate (DMF) reported a significantly lower rate of cortical volume loss with teriflunomide over 24 months (−0.2% vs. −2.94%, *p* = 0.004), as well as a significantly lower percentage of gray matter volume loss at 24 months (−0.44% vs. −3.12%, *p* = 0.015). Teriflunomide also showed a numerically lower rate of whole-brain atrophy over 24 months (*p* = 0.077), although this did not reach statistical significance (18). These results align with a post-hoc analysis of the TOPIC study which confirmed a consistent effect of teriflunomide on reducing loss of cGM volume loss at 24 months (19).

Given that cortical atrophy often begins early in MS and correlates with progression and cognitive impairment, teriflunomide’s ability to reduce cGM loss is particularly noteworthy. It implies that the drug may mitigate the cortical demyelination and neuronal loss that contribute to longer-term disability.

Normal-appearing white matter (NAWM): Teriflunomide’s impact extends to the white matter as well, including NAWM microstructure. A prospective longitudinal study by Zivadinov et al. followed 30 RMS patients with disease duration of 17.7 ± 9.1 years and baseline median EDSS of 3.5 on teriflunomide with advanced MRI techniques (volumetrics and diffusion tensor imaging [DTI]) over 12 months (20). Results showed teriflunomide-treated MS patients had no significant increase in BVL or DTI abnormalities over 1 year when compared to healthy controls (HCs). At baseline, as expected, the MS group had lower WB, GM, and WM volumes and abnormal DTI metrics relative to HC. After 12 months of teriflunomide therapy, MS patients did not deteriorate significantly in GM, WM, or thalamic volume, and their DTI measures remained stable to the point that there were no significant differences between the MS and HC groups at follow-up. Quantitatively, the mean percentage brain volume change in 1 year was −0.56% in the teriflunomide MS vs. − 0.40% in HCs (*p* = 0.305).

The authors concluded that teriflunomide could potentially slow down accumulation of microstructural tissue damage in GM and NAWM and suggested a potential neuroprotective effect. They also noted that no patients had clinical disability progression during that year on therapy. These findings, albeit in a small cohort, suggest that teriflunomide is associated with relative preservation of both gray and white matter integrity.

Thalamic volume: Thalamic atrophy is an early and reliable predictor of cognitive and physical disability in MS. In the aforementioned study, thalamic volume loss was not significantly different from HC (20). Teriflunomide seemed to stabilize thalamic volume over 12 months, preventing the decline typically seen in MS. Concomitantly, there were no differences in thalamic DTI indices between MS and controls over time, indicating that teriflunomide preserves thalamic microstructural integrity (20), supporting teriflunomide’s potential neuroprotective effects on deep GM structures.

### Teriflunomide and disability progression in MS

One of the most clinically meaningful endpoints in clinical trials of MS treatments is their effect of long-term confirmed disability worsening (CDW). Teriflunomide has shown consistent efficacy in reducing disability worsening in both randomized controlled trials and real-world cohorts of patients with RMS and PMS with relapses, with surprising data on recent trials of PMS.

Initial evidence comes from the pivotal phase 3 TEMSO and TOWER trials, which demonstrated that teriflunomide 14 mg significantly reduced the risk of 12-week CDW compared to placebo by approximately 30% in TEMSO over the 108-week core period (21). To further elucidate the underlying mechanism, a *post hoc* analysis of the TEMSO extension study examined the relationship between BVL over 2 years and long-term disability outcomes. Patients with the least BVL (≤0.52%) had markedly lower rates of CDW at Year 7 than those with the greatest BVL (>2.18%) (21). Mediation analysis revealed that BVL alone accounted for 51.3% of teriflunomide’s protective effect on CDW, with additional contributions from suppression of new/enlarging T2 lesions (30.8%) and relapses (38.5%) (21). These findings strongly suggest that teriflunomide’s impact on long-term function is primarily mediated through early preservation of brain volume, more so than inflammatory relapse prevention.

These relationships between imaging markers and functional outcomes have also been validated in real-world settings. The TERICARE study, a large prospective multicenter cohort from Spain, followed 325 RMS patients initiating teriflunomide for up to 2 years. During this period, 79.9% of patients remained free from disability progression, and mean EDSS scores remained stable (EDSS = 1.75 ± 1.5) (22). Notably, this effect was observed across both treatment-naïve individuals and those previously exposed to other DMTs, suggesting a broad applicability of teriflunomide for long-term disease stabilization.

Age is a well-recognized modifier of disability risk in MS, with older patients typically exhibiting more rapid progression and reduced treatment responsiveness. In this context, Berkovich et al. specifically examined the impact of teriflunomide in patients aged 55 years and older who had transitioned from other DMTs. Among 182 PwMS, mean EDSS remained unchanged from teriflunomide initiation (4.5 ± 1.8) to 1 year and increased only slightly to 4.7 ± 1.7 at year two (23). MRI outcomes were favorable, with over 90% of patients showing stable or improved radiological findings during follow-up (23). These results support the feasibility of teriflunomide as a disability-stabilizing agent even in older patient populations with more comorbidities.

The potential for teriflunomide to prevent disability may extend even earlier prior to the onset of clinical MS. The TERIS trial, a multicenter, randomized, double-blind phase 3 study, investigated whether teriflunomide could delay symptom onset in individuals with radiologically isolated syndrome (RIS). Over 96 weeks, the time to first clinical demyelinating event was significantly prolonged in the teriflunomide group compared to placebo, with a 72% relative risk reduction (adjusted hazard ratio: 0.28) (24). Although RIS is not equivalent to RMS, this trial provides compelling evidence that teriflunomide may confer neuroprotection before overt clinical manifestations appear.

While most studies on teriflunomide focus on RMS patients, Ferreira et al. conducted a single-center retrospective analysis to assess the safety and efficacy of teriflunomide in patients with PMS, using glatiramer acetate as a comparator. Among 29 patients treated with teriflunomide (mean age 58, disease duration 16.7 years, EDSS 5.9), no significant differences were observed in sustained EDSS progression or worsening of timed 25-foot walk compared to 30 patients receiving glatiramer acetate, after adjusting for age, sex, and baseline EDSS. Both treatments were well tolerated without serious adverse events (25).

Together, these findings demonstrate that teriflunomide exerts durable effects on disability progression across a wide spectrum of MS from RIS to PMS including older PwMS with longer disease duration. This body of evidence supports teriflunomide’s role as a meaningful DMT with the potential to delay long-term disability accumulation in RMS and PMS.

### Impact of teriflunomide on cognitive performance

Cognitive impairment is a frequent and consequential aspect of MS, affecting processing speed, memory, attention, and executive function in roughly 40–65% of patients (26). Unlike relapses or mobility deficits, cognitive changes can be insidious and are not well reflected by EDSS scores (27). BVL, especially cortical and thalamic atrophy, has been linked to cognitive decline in MS (28).

In the TEMSO Phase III trial and its extension, teriflunomide 14 mg significantly improved the paced auditory serial addition test 3 (PASAT-3) performance over 2 years, with higher cognitive performance maintained through year 5 in patients treated continuously from baseline. Cognitive benefits were greatest among patients with lower BVL, which accounted for 44% of the treatment effect. Compared to relapse activity or lesion load on MRI, BVL had the strongest impact on cognitive outcomes. These findings suggest teriflunomide’s effects on cognition are largely mediated through slowing of brain atrophy (29).

Teriflunomide has also shown the ability to stabilize and improve cognitive performance. In a 96-week real-world study (NEDA-3 Plus), adding symbol digit modalities test (SDMT) to the NEDA criteria, 49% of patients achieved “NEDA-3+” status (no relapses, MRI activity, disability progression, or cognitive decline) while on teriflunomide. Strikingly, all but one patient had no confirmed EDSS progression at 96 weeks, and overall SDMT scores were stable, with significant improvement seen in those who had low baseline SDMT (≤35). Teriflunomide not only prevented physical disability in >94% of this cohort, but also prevented cognitive worsening in the majority, suggesting a protective effect on cognition (30).

However, while the SDMT is sensitive to cognitive change, it primarily assesses processing speed and does not capture all cognitive domains. Prospective cognitive testing supports these findings. Pfaff et al. followed 24 patients with RMS treated with teriflunomide for 2 years using a comprehensive neuropsychological battery (31). Significant improvements were observed in multiple cognitive domains, including long-term verbal memory, reflected by increased scores in the Selective Reminding Test total learning and delayed recall (*p* = 0.018 and *p* = 0.002, respectively), attention and executive function, as measured by improved performance on the Go/No-Go task (*p* = 0.022). No cognitive domain showed evidence of decline over the study period. Additionally, these cognitive gains were accompanied by MRI correlates of structural integrity. Better memory performance was associated with greater preservation of WM volume, particularly within the corpus callosum. Patients who were cognitively impaired at baseline showed significantly more WM atrophy and percentage BVL over the 2-year period compared to cognitively intact patients, reinforcing the link between neurodegeneration and cognitive decline. These findings suggest that relative preservation of white matter integrity under teriflunomide treatment may contribute to observed cognitive benefits (31). Table 1 summarizes key studies on teriflunomide’s impact on brain atrophy and cognition in people with MS (PwMS).

### Comparative efficacy against high efficacy DMTs

In recent years, with the approval of highly effective monoclonal antibodies and next-generation small molecules the MS therapeutic landscape has significantly expanded. While Teriflunomide is often considered a moderate-efficacy oral agent, emerging comparative data provide critical insights into its relative performance, especially when focusing on progression-related outcomes such as brain atrophy, cognitive preservation, and confirmed disability progression (CDP).

Ofatumumab vs. Teriflunomide: The ASCLEPIOS I and II trials directly compared Ofatumumab, an anti-CD20 monoclonal antibody, with Teriflunomide in over 1,800 patients with RMS. Ofatumumab significantly outperformed Teriflunomide in reducing annualized relapse rate (ARR) (rate ratio: 0.49; *p* < 0.001), new/enlarging T2 lesions, and Gd-enhancing lesions. Furthermore, Ofatumumab led to a 34.4% relative risk reduction in 3-month CDP and 32.5% reduction in 6-month CDP versus Teriflunomide (*p* = 0.002 and *p* = 0.01, respectively) (32).

Tolebrutinib vs. Teriflunomide: Tolebrutinib, a CNS-penetrant Bruton tyrosine kinase (BTK) inhibitor, was compared to Teriflunomide in the GEMINI 1 and 2 trials. While Tolebrutinib did not demonstrate superiority in ARR compared to Teriflunomide, it showed a numerical reduction in 6-month CDP (HR 0.71). However, statistical significance was not established due to the hierarchical testing structure in the protocol (33).

Evobrutinib vs. Teriflunomide: A Phase III trial evaluated the safety and efficacy of evobrutinib, a BTK inhibitor, compared to teriflunomide. Evobrutinib did not demonstrate superiority over teriflunomide in reducing the ARR or in any secondary endpoints, including disability progression and MRI measures of focal CNS inflammation (34).

Ublituximab vs. Teriflunomide: The ULTIMATE I and II trials were two parallel, double-blind phase 3 studies that compared intravenous ublituximab (a monoclonal antibody that targets the CD20 antigen) with oral teriflunomide (14 mg) in patients with RMS. Ublituximab significantly reduced ARR compared to teriflunomide in both trials (ULTIMATE I: 0.08 vs. 0.19, rate ratio 0.41; 95% CI, 0.27–0.62; *p* < 0.001; ULTIMATE II: 0.09 vs. 0.18, rate ratio 0.51; 95% CI, 0.33–0.78; *p* = 0.002). MRI findings also favored ublituximab, with a markedly lower number of Gd-enhancing lesions (e.g., 0.02 vs. 0.49 in ULTIMATE I; *p* < 0.001). However, no significant difference in 12-week CDP was observed (HR 0.84; 95% CI, 0.50–1.41; *p* = 0.51). Infusion-related reactions were common (47.7%), and serious infections occurred in 5.0% of ublituximab-treated patients versus 2.9% with teriflunomide (35).

Ocrelizumab vs. Teriflunomide: This observational longitudinal multicenter study in the Swiss MS cohort, was aimed at comparing the effects of Teriflunomide and Ocrelizumab on clinical and MRI endpoints of smoldering activity in 128 RMS patients treated with Teriflunomide and 495 treated with Ocrelizumab. The main outcome was time to progression independent of relapse activity (PIRA), other outcomes included BVL, burden of paramagnetic rim lesions and changes in diffusion tensor imaging (obtained in a subset). Results showed no significant difference in the risk of PIRA (HR for Teriflunomide vs. Ocrelizumab 0.80 [95%-CI:0.40–1.60]; *p* = 0.53). Patients treated with teriflunomide showed a lower annualized rate of BVL (−0.80 [95% CI: −0.91; −0.69 vs. −1.06 [95% CI-1.25; −0.86] *p* = 0.025) and gray matter volume loss (−0.92 [95% CI: −1.05; −0.79] vs. −1.20 [95% CI: −1.43; −0.97]; *p* = 0.035). Results suggest teriflunomide shows similar efficacy to Ocrelizumab on smoldering disease activity with potential greater effect in reducing BVL (36). However, the comparison should be interpreted with caution for the potential confounding effects of the difference in population size between the two groups and differences in prior clinical and treatment histories between the two groups.

## Discussion

In clinical trials, teriflunomide demonstrates consistent long-term benefits on MRI markers of MS progression, notably reducing BVL in RMS, PMS. RIS, and CIS. This effect has been demonstrated in both randomized trials and real-world cohorts. Additionally, it consistently shows a measurable protective effect on specific tissue compartments: it increases NAWM volume, limits cortical GM atrophy, and stabilizes deep GM (thalamic) volume. These MRI findings support the notion that teriflunomide’s impact on MS is more than just WM lesion prevention, it also seems to mitigate the diffuse neurodegenerative damage that contributes to BVL. Such structural MRI-observed benefits provide a mechanistic link to the favorable clinical outcomes on short and longer-term disability observed with teriflunomide. The potential neuroprotective profile of teriflunomide is dose-dependent, with the 14 mg dose yielding greater reductions in lesion activity, atrophy, disability and relapse rates than 7 mg (13, 15, 37).

A decrease in pyrimidine synthesis, with resultant cytostatic effects (4), is the main proposed mechanism of action of teriflunomide (8), but inhibition of DHODH also leads to a host of other lesser-known effects, some of them beneficial while others may not be. As shown in Figure 2, the proposed antioxidant effects may be protective to mitochondria, by way of reduction in oxidative injury to mitochondrial and nuclear DNA, which would reduce apoptosis (38) and may be the main mechanism of action for the neuroprotective effects seen in clinical trials (39). Additionally, improved mitochondrial survival in oligodendrocytes, could consequently support remyelination, be protective of axonal degeneration (7), and reduce TNF-alpha in astrocytes, thereby minimizing inflammation (5). Collectively, these findings may explain the data summarized in this review on decrease in BVL and white matter preservation, which correlated with a decrease in disability progression. Although the collective effects of this drug appear to be on anti-regulated cell death (RCD), further studies are needed to confirm these proposed mechanisms. It is also important to note that inhibition of DHODH has been identified to promote Th1 proinflammatory cells and reduce T-regs, both undesirable effects that could increase inflammation in MS (8). This is supported by the lack of a robust anti-inflammatory effect as seen in the pivotal trials where the cytostatic effect of the drug seemed to only partially override these potentially deleterious effects, resulting in moderate reduction in AAR. Another potential deleterious effect of this drug is that it may enhance ferroptosis (9). However, the role of ferroptosis in MS progression is still unclear.

Several therapies have demonstrated efficacy in slowing disability progression in progressive MS. For example, in the ORATORIO trial, ocrelizumab reduced confirmed disability progression in patients with primary progressive MS (PPMS) (40), whereas in the EXPAND trial, Siponimod reduced confirmed disability progression in patients with secondary progressive MS (SPMS) (41). In a real-world multicenter study to evaluate effects on PIRA in the Swiss MS cohort; 128 RMS patients on teriflunomide and 495 on Ocrevus were followed by a median of 2 and 3 years respectively, teriflunomide was superior to Ocrelizumab in reducing BVL and GM volume loss. This real-world evidence study, despite its limitations (e.g., lower number of patients on teriflunomide) supports teriflunomide’s potential to impact PIRA. Overall, the data summarized in this review supports teriflunomide’s potential to slow disability progression, and in some cases achieve EDSS stabilization, and improve cognitive outcomes, especially when brain atrophy is minimal or low at baseline (21, 29). Evidence also indicates that in CIS, early atrophy predicts long-term disability and that teriflunomide may alter this trajectory when initiated early, with the potential to reduce conversion to CDMS and delay disability milestones.

Teriflunomide’s favorable safety profile, including stable laboratory parameters and good tolerability in older adults, supports its long-term use. Its once-daily oral dosing improves convenience and adherence. Although higher-efficacy therapies may be favored in aggressive highly inflammatory cases, teriflunomide demonstrates findings consistent with potential neuroprotective effects with favorable tolerability for long-term use. Its clinical performance in CDW is comparable to that of BTK inhibitors, and potentially better than Ocrelizumab with a better safety profile.

The cognitive benefits observed with teriflunomide expand its therapeutic role beyond traditional relapse and disability endpoints. Improvements in processing speed, particularly among patients with lower brain atrophy, highlight the importance of volume preservation in maintaining cognitive function. Although often viewed as having moderate anti-inflammatory efficacy, teriflunomide appears to mitigate diffuse neurodegenerative damage, aligning with modern MS strategies that prioritize early intervention and long-term brain health. Importantly, as PIRA is increasingly recognized as a key driver of disability accumulation in MS, additional studies are needed to determine whether the favorable MRI and cognitive effects seen with teriflunomide translate into meaningful effects on progressive MS.

Several limitations should be considered when interpreting the available evidence. First, many of the proposed mechanisms underlying teriflunomide, including neuroprotection and remyelination, remain difficult to confirm. In addition, evidence supporting its role in PMS remains limited, with most available data derived from relapsing MS populations and only a small number of studies specifically evaluating progressive disease, many of which are observational or include relatively small study populations. Cognitive outcomes are likewise subject to important limitations, including practice effects with PASAT, the domain-specific nature of SDMT, and the observational design or limited size of several cohorts. Finally, comparator trial results should be interpreted cautiously, as the inability of investigational therapies to demonstrate superiority over teriflunomide does not by itself establish unique neuroprotective properties of teriflunomide. Therefore, larger prospective studies with formal progression-related endpoints and long-term follow-up are needed to validate these findings and clarify the clinical relevance of the proposed mechanisms.

## Conclusion

Studies of teriflunomide, including recent comparator studies, have revealed somewhat unexpected clinical benefits in key outcomes of disability progression. While teriflunomide is an oral therapy with moderate efficacy in relapse reduction, its proposed antioxidant effects may be protective to mitochondrial function, translating into tissue preservation in MS. In this review with present an important body of evidence supporting a consistent decrease in BVL and on preservation of gray and white matter integrity, including thalamic volume. These structural benefits seem to translate into delayed disability progression and improved cognitive outcomes based on multiple clinical trials, some for progressive disease. Additionally, early and continuous treatment seem to maximize these effects, supporting its use for long-term disease control in the correct setting. These potential neuroprotective effects support teriflunomide as a valuable and well-tolerated option to treating PwMS, especially those with early disease and low inflammatory profile and those with advanced age and signs of progression and/or complications from treatment with high efficacy drugs such anti- B cell therapies. Formal clinical trials in progressive disease are needed to demonstrate its potential benefit on delaying or preventing PIRA, in early RMS patients with good relapse control while on high efficacy DMTs.
