# Supplementary material for: Progression-related outcomes of teriflunomide in multiple sclerosis clinical trials
Source: Front Neurol. 2026 Aug 13;17:1882453. doi: 10.3389/fneur.2026.1882453 (PMC13518363; doi:10.3389/fneur.2026.1882453)
Supplement: Supplementary file 1 [file Table_1.DOCX]

| Title | Authors/Country/Year | Introduction/OBJECTIVE | Method | Conclusion |
| --- | --- | --- | --- | --- |
| Teriflunomide, cognition and MRI: A longitudinal study (1) | Pfaff et al./ France/ 2024 | This study aimed to explore the impact of teriflunomide on comprehensive cognitive function and its MRI correlates, including global and focal brain volume, in RMS patients after two years of treatment. | Twenty-four RMS patients who initiated teriflunomide treatment completed cognitive testing (BCcogSEP battery) and MRI assessments at baseline and after two years. | Both cognitively impaired and preserved RMS patients experienced brain volume loss during teriflunomide treatment, but those with impairment showed significantly greater loss. After two years, the cognitively impaired group improved in SRT mean recall, learning, delayed recall, and Go/no-Go performance. The preserved group improved in SRT delayed recall. No cognitive decline was observed in either group, suggesting that teriflunomide may positively impact cognitive function, particularly verbal memory and inhibition. |
| Effectiveness and safety of switching to teriflunomide in older patients with relapsing multiple sclerosis: A real-world retrospective multicenter analysis (2) | Berkovich et al./ USA/ 2023 | To assess the effectiveness and safety of teriflunomide in older RMS patients who switched from other DMTs. | This retrospective cohort included 182 RMS patients aged ≥55 who switched to teriflunomide (7 or 14 mg) for ≥1 year across 4 U.S. centers. Data spanned 1 year pre- to 2 years post-switch. Effectiveness outcomes included ARR, EDSS, and MRI findings. | MRI outcomes stabilized in most patients, with reduced lesion activity and some showing improvement over 2 years. These findings suggest teriflunomide may slow radiological progression. |
| Effectiveness of teriflunomide on No Evidence of Disease Activity and cognition in relapsing remitting multiple sclerosis: results of the NEDA3PLUS study (3) | Amato et al./ Italy/ 2023 | The NEDA-3 concept was expanded to NEDA-3+ by incorporating cognitive impairment (CI) assessed with the SDMT. The effect of teriflunomide on NEDA-3+ was evaluated in a real-world setting, along with its predictive value for disability progression. | A total of 210 RMS patients in Italy treated with teriflunomide for ≥24 weeks were included. Assessments were conducted at baseline and at weeks 24, 48, 72, and 96. | Teriflunomide showed real-world effectiveness and safety in RMS, especially among treatment-naïve patients or those switching due to tolerability. Cognitive performance remained stable overall, with significant improvement in patients with low baseline SDMT scores. |
| Effects of teriflunomide treatment on cognitive performance and brain volume in patients with relapsing multiple sclerosis: Post hoc analysis of the TEMSO core and extension studies (4) | Sprenger et al./ Germany/ 2022 | To examine the relationship between teriflunomide’s impact on brain volume loss (BVL) and cognitive outcomes. | Cognitive performance (PASAT-3) was assessed in subgroups stratified by 2-year BVL (≤0.52%, >0.52-2.18%, >2.18%). BVL, MRI lesions, and relapses were analyzed as potential mediators of teriflunomide’s effect on cognition. | Teriflunomide 14 mg significantly improved PASAT-3 Z-scores versus placebo by year 2. In the extension, patients with least or intermediate BVL showed greater cognitive gains than those with themost BVL. Mediation analysis indicated that 44% of teriflunomide’s cognitive benefit was mediated by its effect on BVL, suggesting cognition improves largely through BVL preservation. |
| Association of brain volume loss and long-term disability outcomes in patients with multiple sclerosis treated with teriflunomide (5) | Sprenger et al./ Switzerland/ 2020 | To examine whether 2-year BVL in the TEMSO core study predicts long-term CDW at Year 7 in the extension phase. | A total of 709 RMS patients on teriflunomide 14 mg were stratified into tertiles based on 2-year BVL (via SIENA): least (n=221), intermediate (n=357), and most BVL (n=131). CDW at Year 7 was the primary outcome. Mediation analysis evaluated BVL, T2 lesion activity, and relapse rate as contributors to CDW. | Mediation analysis indicated that teriflunomide’s impact on reducing disability progression is largely driven by its effect on BVL. Lower 2-year BVL was linked to a reduced risk of long-term EDSS progression. |
| Slowing of brain atrophy with teriflunomide and delayed conversion to clinically definite MS (6) | Zivadinov et al./ USA /2020 | To explore the effect of teriflunomide on cortical gray matter and whole brain atrophy in CIS patients, and to assess the association between atrophy and risk of conversion to clinically definite MS. | A total of 485 CIS patients were randomized 1:1:1 to placebo (n=159), TFM 7 mg (n=156), or TFM 14 mg (n=170) for up to 108 weeks. In the extension, TFM-treated patients continued their assigned dose, while placebo patients were re-randomized to TFM 7 mg or 14 mg. Brain volume was assessed during years 1-2. | Teriflunomide 14 mg significantly reduced annualized CGM and WB atrophy over years 1-2 vs. placebo, by up to 61.4% and 43%, respectively. Greater brain atrophy was linked to higher CDMS risk, while patients with the least atrophy showed up to a 58% risk reduction with teriflunomide. These findings underscore the clinical relevance of CGM and WB atrophy in CIS and support early TFM use to slow atrophy and delay progression. |
| Comparative effectiveness of teriflunomide and dimethyl fumarate in patients with relapsing forms of MS in the retrospective real-world Teri-RADAR study (7) | Zivadinov et al./ USA/ 2019 | This study compared the real-world effectiveness of TFM versus DMF. The index date was defined as the first use of either drug. Data were categorized into three periods: pre-index (-18 to -6 months), index (-6 months to +2 weeks), and follow-up (9-30 months for clinical data; 9-24 months for MRI). | Anonymized data were collected from 100 RMS patients initiating teriflunomide (n=50) or DMF (n=50). MRI endpoints included volume and count of T2 and Gd+ lesions, as well as changes in whole brain and lateral ventricular volumes. Clinical secondary endpoints were NEDA-3 status and annualized relapse rate (ARR). | Teriflunomide showed superior effectiveness over DMF in reducing whole brain atrophy, with comparable outcomes on other MRI and clinical endpoints. |
| Effect of Teriﬂunomide and Dimethyl Fumarate on Cortical Atrophy and Leptomeningeal Inﬂammation in Multiple Sclerosis: A Retrospective, Observational, Case-Control Pilot Study (8) | Zivadinov et al./ USA/ 2019 | To evaluate the effects of teriflunomide (TFM) and dimethyl fumarate (DMF) on cortical pathology, measured by percentage cortical volume change (PCVC) and leptomeningeal contrast enhancement (LMCE) on MRI. | This retrospective study followed 60 MS patients on teriflunomide and 60 on DMF over 24 months. MRI at baseline, 12, and 24 months assessed PCVC (primary), LMCE foci (secondary), and post-hoc clinical/MRI lesion activity. | Findings suggest that TFM more effectively preserves cortical gray matter volume compared to DMF. |
| 04. Eﬀect of teriﬂunomide on gray and white matter brain pathology in multiple sclerosis using volumetric and diﬀusion-tensor imaging MRI measures (9) | Zivadinov et al. / USA/ 2018 | To investigate teriflunomide’s effect on gray and white matter microstructural pathology in MS, using brain volume and diffusion tensor imaging (DTI) metrics. | Thirty relapsing MS patients and 20 healthy controls were prospectively followed for 12 months with clinical and 3T MRI assessments. | The study found that MS patients on teriflunomide showed similar rates of global, tissue-specific (GM and WM), and regional (thalamic) atrophy as matched HCs over 12 months. Additionally, no significant decline was observed in global or TBSS DTI measures of NAWM and thalamus compared to HCs. |
| Long-Term Outcomes in Patients With Progressive Forms of Relapsing MS Treated With Teriflunomide: Real-World Evidence (10) | Nelson et al./ USA/ 2018 | To present updated long-term disability outcomes in patients with progressive forms of RMS treated with teriflunomide in a real-world setting. | Retrospective data collection from 2 MS centers in the US on 23 patients (22 SPMS, 1 PRMS) treated with teriflunomide (mostly 14 mg) for up to 5 years. EDSS and MRI findings were assessed. | 87% of patients had stable EDSS over the treatment period. Worsening in 3 patients coincided with MRI activity and treatment interruption. Findings support teriflunomide's potential to prevent long-term disability progression in progressive RMS. |
| Magnetic resonance imaging outcomes from a phase III trial of teriflunomide (TEMSO) (11) | Wolinsky et al./ USA/ 2013 | To determine the effects of oral teriflunomide on MS-related pathology as assessed by MRI findings. | In this 108-week trial, 1088 relapsing MS patients were randomized to teriflunomide 7 mg, 14 mg, or placebo once daily. MRI was performed at baseline, 24, 48, 72, and 108 weeks. Primary and key secondary outcomes were ARR and 12-week confirmed disability progression. The main MRI outcome was change in total lesion volume. | Teriflunomide showed sustained benefits on brain MRI measures, with a clear dose effect across most parameters. It significantly reduced relapse rates, slowed disease progression, and limited MRI-defined activity, including new lesions and lesion volume. |
| Randomized Trial of Oral Teriflunomide for Relapsing Multiple Sclerosis (12) | O’Connor et al./ USA/ 2011 | The primary endpoint was annualized relapse rate (ARR), and the key secondary endpoint was 12-week confirmed disability progression. | The study included 363 patients in the placebo group, 366 in the teriflunomide 7 mg group, and 359 in the 14 mg group. | MRI outcomes favored both teriflunomide doses over placebo across multiple measures. Teriflunomide reduced relapses, with the 14 mg dose also slowing disability progression and improving MRI findings, including reduced lesion volume and fewer Gd+ and new/enlarging T2 lesions. However, no significant difference was observed in brain volume loss between groups. |
| Teriflunomide slows BVL in relapsing MS (13) | Radue et al./ Switzerland/ 2011 | This study employed the SIENA method to measure brain volume loss (BVL) in relapsing MS patients receiving once daily teriflunomide as part of the phase 3 TEMSO trial. | In the 2-year phase 3 TEMSO trial, 1088 relapsing MS patients were randomized to receive placebo (n=363), teriflunomide 7 mg (n=366), or 14 mg (n=359) once daily for 108 weeks. SIENA analysis was conducted on 808 patients with quality MRI at baseline and year 2 to assess teriflunomide’s effect on BVL compared to placebo. | Teriflunomide (7 mg and 14 mg) significantly reduced BVL compared to placebo. At years 1 and 2, the 14 mg dose showed 36.9% and 30.6% relative reductions, respectively. These results align with teriflunomide’s known effects on relapse rate, disability, and MRI activity, suggesting a potential neuroprotective role and reinforcing the link between BVL and disability progression in relapsing MS. |

**Supplementary Table 1:** Summary of studies evaluating the effects of teriflunomide on brain atrophy and cognition in PwMS. Abbreviations: BVL, brain volume loss; CDW, confirmed disability worsening; EDSS, Expanded Disability Status Scale; T2, T2-weighted; ARR, annualized relapse rate; Gd+, gadolinium-enhancing; MRI, magnetic resonance imaging; WB, whole brain; CGM, cortical gray matter; CDMS, clinically definite multiple sclerosis; CIS, clinically isolated syndrome; DMF, dimethyl fumarate; PCVC, percentage cortical volume change; LMCE, leptomeningeal contrast enhancement; DTI, diffusion tensor imaging; GM, gray matter; WM, white matter; NAWM, normal-appearing white matter; HC, healthy controls; PASAT-3, paced auditory serial addition test (3-second version); NEDA-3, no evidence of disease activity (relapses, MRI activity, and EDSS progression); SDMT, symbol digit modalities test; PRMS, progressive-relapsing multiple sclerosis; SPMS, secondary progressive multiple sclerosis; RMS, relapsing multiple sclerosis; TFM, teriflunomide; SIENA, Structural Image Evaluation using Normalization of Atrophy.

1.	Pfaff L, Mondino M, Loeb Q, Noblet V, Berthe C, Kremer L, et al. Teriflunomide, cognition and MRI: A longitudinal study. Mult Scler Relat Disord. 2024;90:105793.

2.	Berkovich R, Negroski D, Wynn D, Sellers D, Bzdek KG, Lublin AL, et al. Effectiveness and safety of switching to teriflunomide in older patients with relapsing multiple sclerosis: A real-world retrospective multicenter analysis. Mult Scler Relat Disord. 2023;70:104472.

3.	Amato MP, Bergamaschi R, Centonze D, Mirabella M, Marfia GA, Totaro R, et al. Effectiveness of teriflunomide on No Evidence of Disease Activity and cognition in relapsing remitting multiple sclerosis: results of the NEDA3PLUS study. J Neurol. 2023;270(10):4687-96.

4.	Sprenger T, Kappos L, Sormani MP, Miller AE, Poole EM, Cavalier S, et al. Effects of teriflunomide treatment on cognitive performance and brain volume in patients with relapsing multiple sclerosis: Post hoc analysis of the TEMSO core and extension studies. Mult Scler. 2022;28(11):1719-28.

5.	Sprenger T, Kappos L, Radue EW, Gaetano L, Mueller-Lenke N, Wuerfel J, et al. Association of brain volume loss and long-term disability outcomes in patients with multiple sclerosis treated with teriflunomide. Mult Scler. 2020;26(10):1207-16.

6.	Zivadinov R, Dwyer MG, Carl E, Poole EM, Cavalier S, Briassouli P, et al. Slowing of brain atrophy with teriflunomide and delayed conversion to clinically definite MS. Ther Adv Neurol Disord. 2020;13:1756286420970754.

7.	Zivadinov R, Kresa-Reahl K, Weinstock-Guttman B, Edwards K, Burudpakdee C, Bergsland N, et al. Comparative effectiveness of teriflunomide and dimethyl fumarate in patients with relapsing forms of MS in the retrospective real-world Teri-RADAR study. J Comp Eff Res. 2019;8(5):305-16.

8.	Zivadinov R, Bergsland N, Carl E, Ramasamy DP, Hagemeier J, Dwyer MG, et al. Effect of Teriflunomide and Dimethyl Fumarate on Cortical Atrophy and Leptomeningeal Inflammation in Multiple Sclerosis: A Retrospective, Observational, Case-Control Pilot Study. J Clin Med. 2019;8(3).

9.	Zivadinov R, Bergsland N, Hagemeier J, Carl E, Kolb H, Hojnacki D, et al. Effect of teriflunomide on gray and white matter brain pathology in multiple sclerosis using volumetric and diffusion-tensor imaging MRI measures. J Neurol Sci. 2018;388:175-81.

10.	Nelson F, Lebrun-Frénay C, Boyko A, Thangavelu K, Mandel M, Cavalier S, et al. Long-Term Outcomes in Patients With Progressive Forms of Relapsing MS Treated With Teriflunomide: Real-World Evidence (S8. 004). Neurology. 2018;90(15_supplement):S8. 004.

11.	Wolinsky JS, Narayana PA, Nelson F, Datta S, O'Connor P, Confavreux C, et al. Magnetic resonance imaging outcomes from a phase III trial of teriflunomide. Mult Scler. 2013;19(10):1310-9.

12.	O'Connor P, Wolinsky JS, Confavreux C, Comi G, Kappos L, Olsson TP, et al. Randomized trial of oral teriflunomide for relapsing multiple sclerosis. N Engl J Med. 2011;365(14):1293-303.

13.	Radue EW, Sprenger T, Gaetano L, Mueller-Lenke N, Cavalier S, Thangavelu K, et al. Teriflunomide slows BVL in relapsing MS: A reanalysis of the TEMSO MRI data set using SIENA. Neurol Neuroimmunol Neuroinflamm. 2017;4(5):e390.

References:
